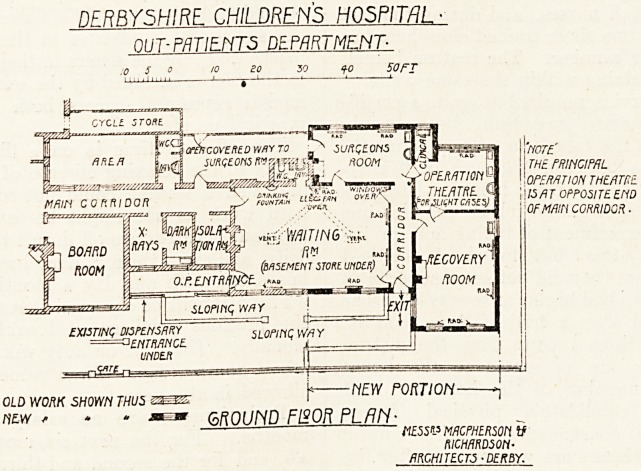# Derbyshire Children's Hospital

**Published:** 1915-05-08

**Authors:** 


					May 8, 1915. THE HOSPITAL 137
HOSPITAL ARCHITECTURE AND CONSTRUCTION.
Derbyshire Children's Hospital.
The accompanying plan shows a small but most
peful addition to the out-patient department of this
hospital.
The waiting hall has been enlarged by the.addi-
tlQn of a room formerly used for isolation, which
kas been superseded by the erection of a new
isolation block. On the north side of the hall a
large surgeons' consulting room has been erecte ,
^rhich communicates on one side with a covere
^ay to the hospital and on the other side wit a
new operation theatre for minor cases, with a small
room for clinical work and a large recovery room.
Three old rooms at the west end of the hall have
now been fitted for use as observation ward, rr-ray
room, and dark room. In the basement the old
dispensary has been enlarged and rearranged to
meet modern requirements.
The alterations were planned by and carried out
under the supervision of Messrs. MacPherson and
Richardson, architects, of Derby.
DERBYSHIRE. CHILDREN2 HOSPITAL
OUT-PATIENTS DEPARTMENT-
;0 J 0 !0 20 30 fO SOFT
y TO _ N SURCEOMS
ItPSOP" TOCI?
p^tk?/; ?r
??? u&fgi Tv'^'-x L THEATRE. V
use.? ove? \ ly^wrMse#
^? \miN6 ?;v
fftf
(BH5?MEIiT .STOKE U/MJflj)
o t~V
EXISTING DISPENSARY S) ,v/? y
=&ENTRANCE * i
UNDER (]
n^n 'I?
tyr?co/?flr
.TO0A7
J
h/y<77Vf
the rmcipfll
" OPERATION THEATRE
\\16fiT OPPOSITE END
OF MAIN CORRIDOR.
-/VEW PORTION-
OLD WORK SHOWN THUS
mw , ' ' 6RQUHD fffQ/f PL/7N-
" MESSV MCPHERSON tf
RICHARDSON-
ARCHITECTS -DERbY.

				

## Figures and Tables

**Figure f1:**